# Prevalence and Co-occurrence of *Candida albicans* and Sexually Transmitted Infections in *Lactobacillus*-Deficient HIV-Negative Adolescent Girls and Young women

**DOI:** 10.1007/s11046-026-01069-2

**Published:** 2026-03-16

**Authors:** Samkelisiwe Beje, Zizipho Z. A. Mbulawa

**Affiliations:** 1 Department of Laboratory Medicine and Pathology, Walter Sisulu University, Mthatha, 5100 South Africa; 2 National Health Laboratory Service, Nelson Mandela Academic Hospital, Fort Gale, Mthatha, 5100 South Africa

**Keywords:** Human papillomavirus, Cervicovaginal microbiota, *Lactobacillus* species, *Candida albicans*, Sexually transmitted infections, Adolescent girls and young women

## Abstract

**Background:**

*Lactobacillus* species are essential for vaginal eubiosis; their depletion often leads to dysbiosis, increasing susceptibility to sexually transmitted infections (STIs). This study investigated the prevalence of *Candida albicans* colonization and its associated factors among HIV-negative adolescent girls and young women (AGYW) in South Africa. Furthermore, STIs associated with *C. albicans* colonization and *Lactobacillus* deficiency were investigated.

**Methods:**

Secondary data analysis was performed on 138 HIV-negative AGYW of Eastern Cape province, South Africa. Secondary data included cervico-vaginal detected *C. albicans*, *Lactobacillus* species (*L. crispatus, L. gasseri* or *L. jensenii*), bacterial vaginosis, human papillomavirus (HPV), *Chlamydia trachomatis*, *Neisseria gonorrhoeae*, *Trichomonas vaginalis*, herpes simplex virus type 1/2 (HSV-1/2), and *Mycoplasma genitalium*, and behavioural questionnaires. Univariate and multivariate logistic regression were performed using GraphPad Prism Version 8.0.1.244.

**Results:**

A proportion of 27.58% were positive for *C. albicans*, and 84.06% tested positive for at least one investigated STI (73.91% for HPV, 31.16% for *C. trachomatis*, 12.32% for *N. gonorrhoeae*, 10.87% for *T. vaginalis*, 7.25% for HSV-1/2, and 5.80% for *M. genitalium*). Frequent sexual intercourse (more than two times within the past 30 days) was associated with higher odds of *C. albicans* (OR: 1.84, 95% CI 1.85–3.80, *p* = 0.036), alcohol consumption (OR: 3.60, 95% CI 1.12–13.23, *p* = 0.038) and recent vaginal discharge syndrome (OR: 2.67, 95% CI 1.06–6.63, *p* = 0.005). Deficiency of Lactobacilli *among C. albicans*-positive AGYW was associated with increased odds of *C. trachomatis* positivity (OR: 4.37, 95% CI 1.05–17.05, *p* = 0.047).

**Conclusion:**

HIV-negative AGYW demonstrated a high burden of STIs, with *C. albicans* positivity associated with alcohol consumption, frequent sexual intercourse, and vaginal discharge syndrome. The absence of Lactobacilli was associated with increased odds of *C. trachomatis* positivity. Further research is recommended using longitudinal designs, larger sample sizes, and diverse populations to better understand these observations.

## Introduction

A healthy vaginal environment depends on the abundance of *Lactobacillus* species [1, 2]. These species are essential regulators of vaginal pH, microbial networks. They produce antimicrobial peptides (AMPs) and a range of bioactive metabolites, including short and medium fatty acids, lactic acid, Hydrogen peroxide (H_2_O_2_), bacteriocins, chitinases, and biosurfactants, that collectively mediate primary defence mechanisms against microbial invasion [3–5]. They effectively compete for adhesion sites in the vaginal epithelium and support the local immune system [4, 5]. However, common changes, such as fluctuations in hormonal levels, can negatively impact the composition of Lactobacilli*,* predisposing women to opportunistic infections and sexual health problems including dry vagina, and vulvovaginal atrophy [6].

Vaginal microbiome marked by lack of Lactobacilli and often has increased aerobic and anaerobic bacteria, dominated by *Gardnerella vaginalis*, which is indicative of vaginal dysbiosis and strongly associated with opportunistic infections [7–9]. *L. crispatus, L. jensenii,* and *L. gasseri* are prominent in maintaining a healthy vaginal microbiome, whereas *L. iners* has shown no significant function in the protective role of the vagina [10–12]. This might be due to the lack of lactic acid and H_2_O_2_ production by *L. iners* [12, 13]. Treatment with Lactobacilli probiotics has proven effective in reducing vulvovaginal candidiasis symptoms such as vaginal dryness, painful swelling, vulvar pruritus and painful urination and preventing vulvovaginal candidiasis recurrence in pregnant women [14]*.*

Candidiasis is likely to occur when the vaginal flora is out of balance or when the immune system is compromised, leading to inflammatory effects that worsen vaginal tissue destruction and increase the possibility of high-risk human papillomavirus (HR-HPV) infection [15]. The changes in the cervicovaginal microbiota, including decreased Lactobacilli and an increase in pathogenic microbes, make the vaginal microbiome prone to persistent HPV [16, 17]. During colonisation, bacteria and pathogens related to STIs produce mucin-degrading enzymes into the vaginal epithelium, causing epithelial cell destruction and a subsequent pro-inflammatory response [18–20]. This phenomenon has been associated with dysbiosis and subsequently the development of cervical cancer [21, 22]. The most common cause of vulvovaginal candidiasis, *C. albicans*, could play a role in the development of cancer [23, 24], alongside high-risk strains of HPV (HPV-16 and HPV-18), which are significant factors in the development of cervical intraepithelial neoplasia (CIN), a precancerous lesion [25, 26]. *C. albicans* is a biofilm-producing fungus [27] that uses this biofilm to escape the immune response, sometimes treatment, and to make complex microbial communities leading to persistent infections [27–29]. In a recent study, *C. albicans* was found in 73% of HPV-16-positive patients and 59.6% of general HR-HPV-positive patients, which could underscore an intricate dysbiosis and tissue damage [15].

Recent findings suggest a high degree of co-occurrence among Candida, bacterial vaginosis, and STIs, particularly *T. vaginalis*, showing a potential syndemic interaction [30]. Bacterial vaginosis showed a strong correlation with concurrent STIs, whereas Candida infections were less frequently associated with *C. trachomatis* and *N. gonorrhoeae* but more common in the presence of *T. vaginalis*. This pattern highlights *T. vaginalis* as a central co-infection in both bacterial vaginosis and Candida contexts, suggesting it could be a major mediator of the imbalance in the vaginal environment [30]. Understanding fungi and STIs co-infections and exploring their relationship is a necessary step that could aid interventions. However, there is a paucity of studies exploring the interplay between fungi and STIs co-infections among AGYW in the Eastern Cape province. Therefore, this study investigated the prevalence of *C. albicans* colonization and its associated factors among sexually experienced HIV-negative AGYW in the Eastern Cape, South Africa. Furthermore, STIs associated with *C. albicans* colonization and *Lactobacillus* deficiency were investigated.

## Materials and Methods

### Ethics Statement

The primary study was approved by the Human Research Ethics Committee at the University of Cape Town, South Africa (HREC: 369/2015), and the Eastern Cape Department of Health (EC_2016RP29_562). Written informed consent was obtained from the legal guardians of participants under 18 years old. All participants aged 18 years and above provided a written informed consent while those less than 18 years provided a written informed assent. No personal identifiers were used during the recruitment of participants. During the primary study recruitment, participants consented for their specimens to be used for further studies provided that the study is approved by Human Research Ethics Committee in South Africa. Therefore, the Walter Sisulu University Human Research Ethics Committee (protocol number: 044/2020) approved the sub-study on *Candida albicans*, STIs, bacterial vaginosis, and *Lactobacillus* species in association with demographic and behavioural factors.

### Study Population and Specimen Collection

This is a retrospective cross-sectional descriptive study among adolescent girls and young women attending high schools who had participated in a parent study, known as the HPV Education Intervention Study, conducted between April and May 2019 [31]. All the adolescent girls and young women were from two high schools in the Chris Hani District Municipality, Eastern Cape Province, South Africa. Study participants and specimen collection were as described elsewhere [31]. Briefly, upon recruitment, participants consented and filled out a detailed questionnaire on demographics, sexual behaviour, alcohol consumption and smoking habits. The current retrospective cross-sectional study included adolescent girls and young women between the ages of 15 and 23 years who were sexually experienced, not menstruation and not pregnant. All participants were asked to provide their HIV status; those who stated negative, rapid HIV testing was performed on the day of recruitment. HIV counselling and testing were performed by a qualified professional nurse or HIV lay counsellor using a rapid antibody screening test using blood from a finger prick. However, the HIV-positive participants were not included in the current study. Self-collected cervicovaginal specimens were collected using the Evalyn® Brush (Rovers® Medical Devices B.V., Oss, Netherlands) as shown on the information sheet provided by the Rovers® Medical Devices and the health professional guidance [31]. The self-collected cervicovaginal specimen was collected by inserting the Evalyn® Brush white brush as far as possible into the vagina after assuming a comfortable stance (either while standing or squatting) and then rotating the plunger 360° five times. The brush was carefully removed from the vagina and capped back after being pulled by the pink plunger into the transparent casing. Brushes with self-collected vaginal specimens were stored at room temperature dry and shipped to the HPV Laboratory at the University of Cape Town, South Africa, for laboratory processing.

### Molecular Detection of HPV, *Lactobacillus* Species, STIs and *Candida Albicans*

The laboratory investigations, processing of the self-collected cervicovaginal specimen and nucleic acid extraction were done as previously reported by Mbulawa et al. [31] and Onywera et al. [32]. Briefly, one millilitre of Digene specimen transport medium (Qiagen, Hilden, Germany) was used to suspend cells from the Evalyn® devise white brush and a total of 400 μl were used for nucleic acid extraction that was conducted using MagNA Pure Compact (Roche Molecular Systems, Inc., Branchburg, NJ, USA) and MagNA Pure Compact Nucleic Acid Isolation Kit (Roche Molecular Systems, Inc., Branchburg, NJ, USA). HPV in extracted nucleic acid was detected using the Roche Linear Array HPV Genotyping Test (Roche Molecular Systems, Inc., Branchburg, NJ, USA) that detects 37 different HPV genotypes (HPV-6, -11, -16, -18, -26, -31, -33, -35, -39, -40, -42, -45, -51, -52, −53, -54, -55, -56, -58, -59, -61, -62, -64, -66, -67, -68, -69, -70, -71, -72, -73, -81, -82, -83, -84, -89, and IS39) and β-globin gene to monitor the quality of the specimen, DNA extraction, amplification and hybridization. The *Candida albicans* was detected in extracted nucleic acid using Allplex™ Candidiasis Assay (Seegene Inc., Seoul, Korea). The *Lactobacillus* species (*Lactobacillus crispatus*, *Lactobacillus gasseri* and/or *Lactobacillus jensenii*) and bacterial vaginosis were also detected from the extracted nucleic acid using the Allplex™ Bacterial Vaginosis plus Assay (Seegene Inc., Seoul, Korea). *Chlamydia trachomatis*, *Neisseria gonorrhoeae* and *Trichomonas vaginalis* were detected using the Allplex™ STI Essential Assay, while herpes simplex virus 1/2 (HSV1/2) was detected using the Allplex™ Genital Ulcer Assays. The Allplex™ Assays were all run on a CFX96™ Real-time PCR Detection System (Bio-Rad)—CFX Manager™ Software-IVD v1.6 and CFX96™ Dx System (Bio-Rad)—CFX Manager™ Dx Software v3.1. Seegene Viewer Software (Seegene Inc., Seoul, Korea) was used to interpret data according to the manufacturer’s instructions.

### Statistical Analysis

*C. albicans*, *Lactobacillus* species, bacterial vaginosis, STI diagnoses, and behavioural questionnaires data on 138 HIV-negative AGYW were initially organised in Microsoft Excel. Further analyses were performed using GraphPad Prism version 10.5.0. Descriptive statistics included medians and interquartile ranges for continuous variables, and frequencies and percentages for categorical variables. Associations between microbial profiles, behavioural factors, and STI outcomes were assessed using logistic regression to estimate odds ratios (ORs) and corresponding 95% confidence intervals (CIs). A significance level of 5% was applied, with *p*-values ≤ 0.05 considered statistically significant. Univariate logistic regression was conducted to identify covariates significantly associated with *Candida albicans* positivity and absence of *Lactobacillus* species. Covariates with *p-*values ≤ 0.05 in univariate analysis were included in a multivariate logistic regression model to adjust for potential confounding and assess interactions between variables.

## Results

The study participant’s characteristics are summarised in Table 1. The median age was 18 years (interquartile range [IQR]: 18–20), with 52.90% of the participants aged between 18 and 20 years. All participants were high school students (Grades 8–12), with the majority in Grades 11 (28.26%) and 12 (29.71%). A total of 80.43% reported never smoking, and 83.33% never consuming alcohol. Regarding sexual practices, most participants reported their sexual debut between ages 16–17 (61.31%), with a median of 2 vaginal sexual encounters in the past month (IQR: 1–3). Overall, 69.47% were currently using contraceptives, and 59.54% used condoms during their most recent sexual activity. Vaginal discharge syndrome with or without itchiness, was reported by 66.66%, while 24.64% reported genital warts, blisters, or ulcers. Based on molecular testing, 27.53% of HIV-negative AGYW were positive for *C. albicans*, 73.91% for HPV, 7.25% for HSV-1/2, 31.16% for *C. trachomatis*, 12.32% for *N. gonorrhoeae,* 10.87% for *T. vaginalis*, and 5.80% for *M. genitalium*. The majority of HIV-negative AGYW (84.06%) were positive with at least one of these investigated STIs (HPV, HSV-1/2, *C. trachomatis, N. gonorrhoeae, T. vaginali,* or *Mycoplasma genitalium*, Table 1).

**Table 1:** Characteristics of HIV-negative sexually experienced AGYW participants

Baseline characteristics	All participants, N = 138	
*Age (years); median (IQR)*	18	(18–20)
*Age group (years); n (%)*		
15–17	29	21.01%
18–19	73	52.90%
20–23	36	26.09%
*Level of education, grade; n (%)*		
Grade 8	8	5.80%
Grade 9	13	9.42%
Grade 10	37	26.81%
Grade 11	39	28.26%
Grade 12	41	29.71%
*Ever smoke; n (%)*		
Yes	27	19.57%
No	111	80.43%
*Sexual debut, years; median (IQR)*	16	(15–17)
*n (%)*^*Σ*^		
≤ 15 years	53	36.69%
16–17 years	84	61.31%
*Number of lifetime sexual partners; median (IQR)*^*Σ*^	2	(1–3)
*Number of sexual partners past 3-months; median (IQR)*^*Σ*^	1	(1–1)
*Frequency of vaginal sexual intercourse in the past 1 month; Median (IQR)*	2	(1–3)
*Consume alcohol, n (%)*		
No	23	16.67%
Yes	115	83.33%
*Ever pregnant, n (%)*		
No	105	76.09%
Yes	33	23.91%
*Currently on contraception*^*Σ*^*, n (%)*		
No	40	30.53%
Yes	91	69.47%
*Condom use during last intercourse*^*Σ*^*, n (%)*		
No	78	59.54%
Yes	53	40.46%
*Vaginal discharge syndrome or itching, n (%)*		
No	46	33.33%
Yes	92	66.66%
*Genital warts, blisters or ulcers, n (%)*		
No	104	75.36%
Yes	34	24.64%
*HPV, n (%)*		
Negative	36	26.09%
Positive	102	73.91%
HR-HPV	77	55.80%
*HSV1/2, n (%)*		
Negative	128	92.75%
Positive	10	7.25%
*C. albicans¸n (%)*		
Negative	100	72.46%
Positive	38	27.54%
*C. trachomatis, n (%)*		
Negative	95	68.84%
Positive	43	31.16%
*N. gonorrhoeae, n (%)*		
Negative	121	87.68%
Positive	17	12.32%
*T. vaginalis,* n (%)		
Negative	123	89.13%
Positive	15	10.87%
*M. genitalium,* n (%)		
Negative	130	94.20%
Positive	8	5.80%
Any STI*, n (%)		
No	22	15.94%
1 STI	54	39.13%
2 STIs	48	34.78%
3–4 STIs	14	10.14%

IQR, Interquartile range; n (%), Column guide—Number of participants followed by percentage; N, Total number of participants enrolled; HPV, Human Papillomavirus; HSV1/2, herpes simplex virus types 1 or 2.;STIs, Sexually transmitted infections; ^Σ^, Some participants are non-responders in this section; Therefore, the total number of participants decreased from the original 138; *Any of the investigated STIs (HPV, HSV-1/2, *C. trachomatis, N. gonorrhoeae, T. vaginali,* or *Mycoplasma genitalium*)

### Association of *C. Albicans* With Behavioural Factors and STI Among HIV-Negative AGYW, Regardless of *Lactobacillus* Species Status

In univariate analysis presented in Table 2, HIV-negative AGYW who had more than two times frequency of vaginal sexual intercourse within the past 30 days had nearly double the odds of testing positive for *C. albicans* (OR: 1.84, 95% CI 1,85–3.80, *p* = 0.036). Those who consume alcohol had over three times the odds of being positive with *C. albicans* (OR: 3.60, 95% CI 1.12–13.23, *p* = 0.038). Having 3–4 of the investigated STIs had increased odds of testing positive for *C. albicans* (OR: 3.14, 95% CI 1.23–8.46, *p* = 0.029) and those who had experienced VDS or itchiness in the past 0–30 days (OR: 2.67, 95% CI 1.06–6.63, *p* = 0.005). When multivariate analysis was conducted among the significant variables from the univariate analysis, none were statistically significant.

**Table 2:** Association of *C. albicans* with behavioural factors and STIs among HIV-negative AGYW regardless of *Lactobacillus* species status (Univariate analysis)

Characteristics	Total	*Candida albicans*				OR (95% CI)	*p *value
		Positive		Negative			
	N = 138	n	%	n	%		
*Age group (years)*							
15–17	29	7	24.14	22	75.86	Ref	
18–19	73	20	27.40	53	72.60	1.14 (0.43–2.88)	> 0.999
20–23	36	11	30.56	25	69.44	1.26 (0.46–3.37)	0.790
*Level of education, grade*							
Grade 8	8	2	25.00	6	75.00	Ref	
Grade 9	13	4	30.77	9	69.23	1.23 (0.21–7.57)	> 0.999
Grade 10	37	8	21.62	29	78.38	0.86 (0.18–4.67)	> 0.999
Grade 11	39	10	25.64	29	74.36	1.03 (0.23–5.40)	> 0.999
Grade 12	41	14	34.15	27	65.85	1.37 (0.26–6.97)	> 0.999
*Ever smoke*							
No	111	30	27.03	81	72.97	Ref	
Yes	27	8	29.63	19	70.37	1.10 (0.47–2.72)	0.821
*Sexual debut, years *^*Σ*^	(N = 137)						
≤ 15 years	53	14	26.42	39	73.58	Ref	
16–17 years	84	24	28.57	60	71.43	1.08 (0.51–2.22)	> 0.999
*Number of lifetime sexual partners* ^Σ^	(N = 131)						
1	38	8	21.05	30	78.95	Ref	
2–3	75	22	29.33	53	70.67	1.39 (0.59–3.32)	0.378
4–6	18	7	38.89	11	61.11	1.85 (0.61–5.34)	0.365
*Number of sexual partners past 3-months* ^Σ^	(N = 131)						
0	23	5	21.74	18	78.26	Ref	
1	90	25	27.78	65	72.22	1.28 (0.48–3.33)	0.792
2–4	18	7	38.89	11	61.11	1.79 (0.47–6.19)	0.514
*Vaginal sexual intercourse frequency past 1-month *^*Σ*^	(N = 133)						
0–1	65	13	20.00	52	80.00	Ref	
≥ 2	68	25	36.76	43	63.24	1.84 (1.85–3.80)	0.036
*Consume alcohol*							
No	23	2	8.70	21	91.30	Ref	
Yes	115	36	31.30	79	68.70	3.60 (1.12–13.23)	0.038
*Ever pregnant*							
No	105	29	27.62	76	72.38	Ref	
Yes	33	9	27.27	24	72.73	0.99 (0.40–2.28)	> 0.999
*Currently on contraception*^*Σ*^	(N = 131)						
No	40	14	35	26	65.00	Ref	
Yes	91	23	25.27	68	74.73	0.722 (0.35–1.49)	0.294
*Condom use during last intercourse*^*Σ*^	(N = 131)						
No	78	25	32.05	53	67.95	Ref	
Yes	53	12	22.64	41	77.36	0.71 (0.32–1.48)	0.323
*Any HPV*							
Negative	36	5	13.89	31		Ref	
Positive	102	33	32.35	69	67.65	2.33 (0.89–5.83)	0.049
*Any HPV*							
Negative	36	5	13.89	31	86.11	Ref	
Positive single type	26	9	34.62	17	65.38	2.49 (0.71–7.44)	0.070
Positive multiple types	76	24	31.58	52	68.42	2.27 (0.81–5.81)	0.064
*HR-HPV*							
Negative for any HPV	36	5	13.89	31	86.11	Ref	
Negative but LR-HPV positive	25	9	36.00	16	64.00	2.59 (0.73–7.77)	0.064
Positive	77	24	31.17	53	68.83	2.24 (0.80–5.74)	0.065
*C. trachomatis*							
Negative	95	24	25.26	71	74.74	Ref	
Positive	43	14	32.56	29	67.44	1.29 (0.63–2.65)	0.414
*N. gonorrhoeae*							
Negative	121	30	24.79	91	75.21	Ref	
Positive	17	8	47.06	9	52.94	1.90 (0.76–4.87)	0.079
*T. vaginalis*							
Negative	123	35	28.46	88	71.54	Ref	
Positive	15	3	20.00	12	80.00	0.70 (0.21–2.57)	0.76
*M. genitalium*							
Negative	130	34	26.15	96	73.85	Ref	
Positive	8	4	50.00	4	50.00	1.91 (0.61–5.94)	0.216
*HSV1/2*							
Negative	128	35	27.34	93	72.66	Ref	
Positive	10	3	30.00	7	70.00	1.10 (0.31–4.13)	> 0.999
*Any STI*							
Negative	22	4	18.18	18	81.82	Ref	
1 STI	54	12	22.22	42	77.78	1.22 (0.37–3.77)	0.767
2 STIs	48	14	29.17	34	70.83	1.60 (0.51–4.86)	0.391
3–4 STIs	14	8	57.14	6	42.86	3.14 (0.80–10.54)	0.029
*Bacterial vaginosis*							
Normal	57	16	28.07	41	71.93	Ref	
Intermediate	18	6	33.33	12	66.67	1.19 (0.39–3.58)	0.768
Positive	63	16	25.40	47	74.60	0.90 (0.40–2.05)	0.837
*Lactobacillus* species							
Negative	72	16	22.22	56	77.78	Ref	
Positive	66	22	33.33	44	66.67	1.75 (0.84–3.83)	0.182
*Experienced VDS or itching*							
No	46	8	17.39	38	82.61	Ref	
Yes, 0–30 days	41	19	46.34	22	53.66	2.67 (1.06–6.63)	0.005
Yes, 1–6 months	21	8	38.10	13	61.90	2.19 (0.73–6.21)	0.120
Yes, ≥ 6 months	30	3	10.00	27	90.00	0.58 (0.16–2.17)	0.511
*Ever had genital ulcers, blisters/warts*							
No	104	28	26.92	76	73.08	Ref	
Yes, 0–30 days	23	6	26.09	17	73.91	0.97 (0.37–2.59)	> 0.999
Yes, 1–6 months	3	1	33.33	2	66.67	1.24 (0.09–8.56)	> 0.999
Yes, ≥ 6 months	8	3	37.50	5	62.50	1.39 (0.38–5.07)	0.683

Ref, Reference; OR, Odds ratio; CI Confidence interval; *p*-Value, Probability value; IQR, Interquartile range; n (%), Column guide—Number of participants followed by percentage; N, Total number of participants enrolled; HPV, Human papillomavirus; LR-HPV, Low-risk human papillomavirus; HR-HPV, High-risk human papillomavirus; HSV1/2, herpes simplex virus types 1 or 2. STIs; Sexually transmitted infections; VDS, Vaginal discharge syndrome; ^Σ^, Some participants are non-responders in this section; Therefore, the total number of participants decreased from the original 138. The *p*-value bolded indicates significance

### STIs and Clinical Outcomes According to *Candida Albicans *and *Lactobacillus* Species Among Hiv-Negative AGYW

Overall, 11.59% (16/138) of participants were positive for *C. albicans* and negative for *Lactobacillus* spp while 15.94% (22/138) were positive for both *C. albicans* and *Lactobacillus* spp. In Table 3univariate analysis presented in Table 3, the *C. albicans* positive and Lactobacilli-negative HIV-negative AGYW showed higher odds for testing positive for *C. trachomatis* when compared with individuals who were positive for both *C. albicans* and *Lactobacillus* species (OR: 4.37, 95% CI 1.05–17.05, *p* = 0.047). When similar analysis was conducted on other STIs, an increased odds of testing positive for HPV, *T. vaginalis,* or *Mycoplasma genitalium* were observed but statistically significant (Table 3). When multivariate analysis was conducted among the significant variables from the univariate analysis, none were statistically significant.

**Table 3:** Sexually transmitted infections (STIs) and clinical outcomes among *Candida albicans-positive* and Lactobacilli*-negative* HIV-negative AGYW (univariate analysis)

Characteristics	Total	*C. albicans* pos & *Lactobacillus* neg, N = 16		*C. albicans* pos & *Lactobacillus* pos, N = 22			OR (95% CI)	*p *value
		n	%	n	%			
*Any HPV*								
Negative	5	1	20.00	4	80.00		Ref	
Positive	33	15	45.45	18	54.55		3.33 (0.44–43.14)	0.374
*C. trachomatis*								
Negative	24	7	29.17	17	70.83		Ref	
Positive	14	9	64.29	5	35.71		4.37 (1.05–17.05)	0.047
*N. gonorrhoeae*								
Negative	30	14	46.67	16	53.33		Ref	
Positive	8	2	25.00	6	75.00		0.38 (0.07–2.04)	0.426
*T. vaginalis*								
Negative	35	14	40.00	21	60.00		Ref	
Positive	3	2	66.67	1	33.33		3.00 (0.32–45.23)	0.562
*M. genitalium*								
Negative	34	13	38.24	21	61.76		Ref	
Positive	4	3	75.00	1	25.00		4.85 (0.64–65.41)	0.291
*HSV1/2*								
Negative	35	15	42.86	20	57.14		Ref	
Positive	3	1	33.33	2	66.67		0.67 (0.04–6.21)	> 0.999
*Any STI*								
Negative	4	1	25.00	3	75.00		Ref	
Positive	34	15	44.12	19	55.88		2.37 (0.32–32.56)	0.625
1 STI	12	3	25.00	9	75.00		1.00 (0.10–16.91)	> 0.999
2 STIs	14	7	50.00	7	50.00		3.00 (0.35–43.77)	0.588
3–4 STIs	8	5	62.50	3	37.50		5.00 (0.46–75.79)	0.546
*Experienced VDS or itching*								
No	8	1	12.50	7	87.50		Ref	
Yes	30	15	50.00	15	50.00		7.00 (0.93–83.31)	0.106
*Ever had genital ulcers, blisters/warts*								
No	28	11	39.29	17	60.71		Ref	
Yes	10	5	50.00	5	50.00		1.55 (0.35–6.78)	0.713

OR, Odds ratio; CI, Confidence interval; *p*-Value, Probability value; IQR, Interquartile range; n (%), Column guide—Number of participants followed by percentage; N, Total number of participants enrolled; HPV, Human papillomavirus; LR-HPV, Low-risk human papillomavirus; HR-HPV, High-risk human papillomavirus; HSV1/2, herpes simplex virus types 1 or 2; STIs, Sexually transmitted infections; VDS, Vaginal discharge syndrome; The *p*-value bolded indicates significance; Ref, Reference.

## Discussion

This study investigated the prevalence and associated factors of vaginal *Candida albicans* and its effects, in the absence of *Lactobacillus* spp., on STIs among sexually experienced HIV-negative AGYW of Eastern Cape province, South Africa. To the best of our knowledge, this is the first study to report on this topic among AGYW in this province. Although the global age-standardised rate of STI incidence nearly doubled over the past two decades, rising from 26.7 million in 1990 to 55.8 million in 2021 [33], it has become evident that women, particularly adolescents and teenagers, are more susceptible to STIs [34]. In the current study, STIs, including HPV, *C. trachomatis*, *N. gonorrhoeae*, *T. vaginalis*, HSV-1/2, and *M. genitalium*, were detected among HIV-negative AGYW, with 84.06% testing positive for at least one of these STIs. Several studies have shown that the prevalence of STIs among adolescents tends to be higher than that of adults (62.8% vs. 34.0% in the Western Cape; 42.5% vs. 16.4% in KwaZulu-Natal) [38]. Similar observations have been reported in Eastern Cape province, where Taku et al. reported lower prevalence of HR-HPV (32.2%), HSV-1/2 (5.9%), *C. trachomatis* (2.4%)*, N. gonorrhoeae* (1.5%), and *M. genitalium* (1.5%) among adults than the one observed in the current study, except for *T. vaginalis* (15.9%) [22]. This suggests that even HIV-negative AGYW may face a high STI burden, although some evidence has shown that HIV-positive women have higher chances of HR-HPV and STI co-infections [35, 36]. The high prevalence of STIs among HIV-negative individuals may indicate that this group is also at an increased probability of HIV acquisition [37] and new sexual relationships may increase probability of STI transmission [39]. A high burden of HPV was observed among AGYW in Tanzania, particularly at the onset of sexual activities [40].

The current study suggests that individuals who have experienced vaginal discharge syndrome or itchiness within the last 0–30 days had twice the odds of testing positive for *C. albicans*. Bacterial vaginosis had been identified as the primary cause of vaginal discharge syndrome; however, *C. albicans* infection is also a significant contributor [42]. Similarly, HSV-2 and syphilis are associated with genital ulcers [43]. In cases of STI co-infections, which were also observed in this study, these signs may be more severe, accompanied by excessive genital inflammation, destruction of the epithelial lining in the vaginal tract [37], persistent HPV, and increased susceptibility to cervical intraepithelial neoplasia and cervical cancer development [21, 22]. Testing positive for *C. albicans* was associated with a 77% probability of persistent HPV infection in women who were previously infected, according to the ensuing longitudinal analysis [41]. Recent findings among women in Namibia indicate that 38% of those who tested positive for any *Candida* species also tested positive for STIs. This included 4.5% with *N. gonorrhoeae*, 6.4% with *C. trachomatis*, and 27% with *T. vaginalis*. Individuals who tested positive for *C. trachomatis* were more likely to test negative for Candida, with a rate of 6.4% compared to 16% for those without *C. trachomatis* [30].

In current findings, individuals who consume alcohol and engaged frequently (more than twice) in vaginal sexual intercourse per month were associated with twice the odds of testing positive for *C. albicans*. Sexual behaviours, smoking, alcohol consumption, and psychosocial stress have been strongly associated with increased susceptibility to STIs [44, 45]. In this study, the absence of *Lactobacillus* species in individuals who tested positive for *C. albicans* was significantly associated with nearly three times the odds of testing positive for *C. trachomatis*. Several studies have highlighted the beneficial effects of *Lactobacillus* species against various pathogens [10–12]. Mechanistically, these *Lactobacillus* species can reduce hyphal formation and inhibit early biofilm development, both of which are critical factors in the pathogenicity of Candida [46, 47]. However, it is important to note that not all *Lactobacillus* species exhibit these protective effects, only *Lactobacillus crispatus*, *Lactobacillus gasseri* and/or *Lactobacillus jensenii* had been shown to demonstrated these effects [47].

Some aspects of the study may have limited the study, this includes questionnaire data where participants were asked to recall past events, such as sexual behaviour, habits related to alcohol and smoking, and history and signs of vaginal infections. Self-reported data from participants can be subject to recall bias or social desirability bias. Memories can be inaccurate, and participants may struggle to accurately recall these events, which can lead to misreporting. It is also important to note that it is a norm in many societies and cultures, especially in the Eastern Cape province of South Africa, not to freely talk about sexually related activities and events [48]. As a cross-sectional study, this study drew conclusions from observations made at one point in time. Thus, it did not establish cause-and-effect relationships between behavioural, habitual, or medical history variables and infection with STIs, nor between the occurrence of *C. albicans* and the presence or absence of *Lactobacillus* species. However, this study was able to identify link between these variables. The study was conducted in only two high schools in a specific district (Chris Hani District Municipality, Eastern Cape Province), which limits the generalizability of the findings to other regions of South Africa or to different populations of AGYW. In addition, the current study’s generalization and reliability could also be affected by the limited sample size. Those who were menstruating or pregnant were excluded, which means the findings may not be generalizable to all sexually experienced AGYW. Lastly, the specimen was self-collected by the participants with guidance; self-collection carries a risk of improper technique, which could lead to inadequate or contaminated specimens, potentially affecting the accuracy of laboratory results.

In summary, Eastern Cape province of South Africa HIV-negative AGYW demonstrated a high burden of STIs, with *C. albicans* positivity associated with alcohol consumption, frequent sexual intercourse, and vaginal discharge syndrome. The absence of Lactobacilli among *C. albicans* positive AGYW was associated with increased odds of *C. trachomatis* positivity. Further research is recommended using longitudinal designs, larger sample sizes, and diverse populations to better understand these associations and improve generalizability.

## Data Availability

The data is available on request from the corresponding author.
